# Influence of Various Chemical Surface Treatments, Repair Materials, and Techniques on Transverse Strength of Thermoplastic Nylon Denture Base

**DOI:** 10.1155/2020/8432143

**Published:** 2020-09-09

**Authors:** Ghassan Abdul-Hamid Naji

**Affiliations:** Department of Prosthetic Dentistry, College of Dentistry, University of Baghdad, Bab-Almoadham, P.O. Box 1417, Baghdad, Iraq

## Abstract

The process of repairing the fractured nylon denture bases and addition of acrylic teeth to the previously worn nylon denture bases has not been widely studied. This study aims to assess the transverse strength of nylon denture bases repaired by various resin materials, different curing techniques, and types of surface treatments. *Materials and Methods*. One hundred fifty thermoplastic nylon denture base samples were fabricated using plastic patterns measuring 65 × 10 × 2.5 mm (length, width, and thickness, respectively). These samples were then divided into three equal groups. Fifty samples were repaired by microwave heat-polymerization, fifty samples were repaired using the Ivomate autopolymerization, and the other fifty were repaired using light-polymerized acrylic resin. Each of these three groups was further divided into five subgroups of ten samples based on the type of surface treatment. The samples in the control group did not undergo any surface treatment, and the other four groups were chemically surface treated with monomer, acetone, ethyl acetate, and isopropanol, respectively. A three-point bending test was used to calculate the transverse strength values of the samples. Fourier transform infrared (FTIR) analysis was conducted to determine the component of functional groups between the polyamide nylon base and poly(methyl-methacrylate) PMMA repair materials. A polarizing microscope was utilized to investigate the mode of failure at the fracture surfaces. *Results*. The collected data were analyzed with one-way ANOVA and Sidak's multiple comparison test to show the differences among different groups. For surface treatments, the highest transverse strength values were obtained by monomer-treated samples (18.29 N/mm^2^); however, the lowest values were obtained in non-surface treated samples (5.58 N/mm^2^). While for repair techniques, the highest transverse strength values were obtained by microwave processing, followed by Ivomate and then the light-cured polymerization. The means were found to be significant (*p* < 0.001). FTIR analysis shows the presence of hydrogen bonding which is due to the ester and amid groups which enhance the bond strength of the surface-treated samples. The interface of the polarizing microscope images revealed a cohesive fracture within repair materials rather than the adhesive nature. *Conclusion*. The microwave-polymerized resin was considered as the most effective repair technique along with monomer chemical etchant which creates a tight adhesion between PMMA and nylon denture base in comparison to other groups.

## 1. Introduction

Polyamide nylon base is a family of condensation polymers produced from the reaction of a diacid with a diamine monomer to form a variety of polyamides whose physical and mechanical properties are based on the bonding links between the acid group and amine group [1]. Recently, the use of lightweight denture fabrication materials such as thermoplastic nylon has gained a lot of interest. With the technological advancements of prosthetic dentistry, there is a demand to identify the most suitable bonding techniques that can be utilized while performing repairs of these new materials. Optimum performance of the material after such repairs is a key consideration.

Precise and accurate denture bases are fabricated using the thermoplastic resins when prepared utilizing the injection moulding technique which leads to less polymerization shrinkage. Creep resistance, higher fatigue resistance, and dimensional stability are some of the advantages of thermoplastic resins over conventional powder and liquid systems [2–4]. Nylon is a generic name for certain types of thermoplastic polymer belonging to a class of polyamide [5]. Nylon resins have gained popularity and are widely being accepted in clinical practice as a suitable choice of denture base materials. An esthetically favorable outcome, higher elasticity, and sufficient transverse strength than the conventional heat-polymerizing resins advocate its use [5, 6]. Also, thermoplastic nylon resins are a suitable alternative for patients who are allergic to conventional metals and free monomer as studies have revealed a little or almost no free monomer releaser with the use of nylon bases [7].

Various processing techniques such as heat-polymerized, autopolymerized, light-polymerized, or microwave-polymerized acrylic resins have been used to repair the fractured dental prosthesis [8]. However, the autopolymerizing acrylic resin has been commonly utilized for repairing the fractured denture base and chipped artificial teeth. Regarding the surface treatment, a silica-coating by Rocatec® followed by silane coupling to improve the adhesion properties by using the sandblasting method has been used [9, 10].

The adhesion between the nylon base and repair materials can be further enhanced by surface treatment of the denture bases using different chemicals. These chemicals etch the surface and modify the morphology and chemical properties of the denture base [11]. Methyl methacrylate (MMA) has been commonly used for treating the fractured surfaces of the denture bases [12]. However, organic solvents such as acetone, ethyl acetate, isopropanol, toluidine [13], chloroform [14], and methylene chloride [15–17] have also been used to perform the surface treatment. Chemical surface treatment has been reported to raise the bond strength between the resin used for repair and the denture base [16]. The air blast technique is another surface treatment method, which involves the collision of the accelerated silica-coated alumina particles with the surface which results in the microscopic melting of the treated surface. This process allows the silica-coated alumina particles to penetrate and form an adhesive bond with the surface [18]. The adhesive bond exhibits satisfying strength on initial assessment; however, the aging process has revealed poor outcomes [9].

Solvent-assisted bonding is an effective method for repairing thermosetting acrylic resins [19]. Polyamides are generally stable and resistant when exposed to chemical insults. However, the presence of amide groups (-NHCO-) in a solvent makes polyamide prone to absorb water or other solvents and to form hydrogen bonds [20]. Traces of polar molecules in solvents cause plasticization of the polyamide matrix [21]. The plasticization of polyamides disrupts the network of hydrogen bonds which enhances the chain mobility [20]. Propionic acids, acetic acids, and butyric acids promote the adhesion through hydrogen bonding. Also, they cause hydrolysis that breaks the crosslinks and provides swelling generally similar to those of organic solvents [22].

Koodaryan and Hafezeqoran [23] revealed that the surface treatment of polyamide denture base with 5% acetic acid in aqueous ethanol (30/70 by volume) for 10 minutes may be an efficient and cost-effective method for increasing the shear bond strength to the autopolymerized reline resin.

This study aimed to evaluate the physical and mechanical properties for the adhesive bond of the thermoplastic nylon denture base using different resin materials, curing techniques, and chemical surface treatments.

## 2. Materials and Methods

### 2.1. Sample Grouping

The materials used in this study are listed in Table 1. A total of 150 sample bases of thermoplastic nylon resin were prepared. Fifty sample bases were repaired by microwave (micro) heat-polymerized resin, fifty were repaired using Ivomate (Ivo) autopolymerized resin, and fifty sample bases were repaired using light-polymerized (light) acrylic resin materials. Each of the three groups was further subdivided into five equal groups based on different chemicals used for surface treatment. These included the control group (Cont) without any surface treatment and four chemical groups which were monomer (Mon), acetone (Acet), ethyl acetate (Eth), and isopropanol (Iso).

### 2.2. Sample Preparation

Plastic patterns measuring 65 ± 0.3 (length) × 10 ± 0.03 (width) × 2.5 ± 0.03 mm (thickness) with wax sprues were prepared for the transverse strength test, according to the Specification No. 12 of the American Dental Association [24]. After wax elimination, the surface of the mould was painted with a separating medium (cold mould seal) using a brush tip. The polyamide capsule was placed in the metal ring heater of the injection machine for 12 minutes, and the temperature was raised to 288°C under the pressure of 0.1 MegaPascal (MPa) to prepare the polyamide for injection [25]. Meanwhile, the metal flask was screwed tightly and placed in a hot oven at 75°C for 12 minutes [26].

The prepared nylon sample bases were stored in distilled water at 37°C for 24 hours, following which the samples were cut in half (3 mm width) with a disk bur at a 45° bevel using metal holding device. The cutting process was performed under running water guided via a standardized positional jig. The butt joint surfaces were prepared with a coarse stone bur for mechanical retention. The chemical surface treatment varied based on each chemical group where a swab was used to apply the monomer for 60 seconds [27], acetone for 30 seconds, and ethyl acetate for 120 seconds, while isopropanol for 5 seconds [13]. The sample bases were then left to dry for 30 seconds [10]. The samples were repositioned into the same stone indices in such a way that a 3 mm gap existed between the two sections of the sample [28].

### 2.3. Repair Technique

For the light-curing repair technique, the powder and liquid were mixed according to the manufacturer instructions (a mixing ratio of 1 g : 0.5 mL) until it reached the dough stage. The dough was then adapted in the stone mould by finger pressure having the same dimensions as the original specimens. The curing cycle was 470 nm wavelength for 10 minutes using a light-curing unit (Yeti Dentalprodukte, Germany) [27]. For microwave curing repair, the heat polymerized powder and liquid were mixed according to the manufacturer's instructions (a mixing ratio of 3 : 1 by volume) till it reached a dough stage. Then, the dough was packed into the stone mould inside the microwave flask (Tecnoflask, Intro kit, Germany) and processed with 500 W power output for 3 minutes [29] using a microwave oven (BK MD 1500, Beko, Istanbul, Turkey). While for Ivomate curing repair, the autopolymerized powder and liquid were mixed according to the manufacturer's instructions (a mixing ratio of 2.5 : 1 by volume), and the dough material was packed into the stone mould and allowed to cure at 45°C for 15 minutes under the pressure of 2 bars [27] using Ivomate device (Palamat Practic ELT, Heraeus Kulzer, Wehrheim, Germany) as presented in Table 1. Then, the samples were bench-cooled for 30 minutes. The surfaces of each sample were trimmed using acrylic and stone burs followed by manual grinding using 600 grit silicon carbide paper to remove any excess material and to obtain the final dimension. The samples were then cleaned and stored in distilled water at 37°C for 48 hours.

### 2.4. Transverse Strength Test

A three-point bending test was applied for testing the transverse strength of all samples using a universal testing machine (Model 1190, High Way Combe Bucks, UK). The device supplies a loading roller and a pair of supporting rollers (3.2 mm diameter) placed at a span length of 50 mm. This test was conducted at a cross-head speed of 5 mm/min. The load was gradually and perpendicularly directed to the center of the repaired area until a fracture occurred. The ultimate fracture load was recorded for each sample, and the transverse strength was calculated using the following equation [13]:

(1) $$\begin{array}{c} \text{TS}=\frac{3Wl}{2bd^{2}}, \end{array}$$

where TS is the transverse strength (Newton (N)/mm^2^), *W* is the load causing fracture (N), *l* is the distance between supporting wedges (50 mm), *b* is the width of the sample (10 mm), and *d* is the thickness of the sample (2.5 mm).

### 2.5. Fourier Transform Infrared Spectroscopy Test

Regarding the curing technique and resin materials, different samples from each group were scratched and attenuated for total reflection Fourier transform infrared spectroscopy analysis (ATR-FTIR tensor 27, Bruker, Germany) to detect the component of chemical functional groups formed on the surface of the polyamide nylon base and poly(methyl-methacrylate) (PMMA) repair materials which were processed by different polymerization techniques. The infrared spectra of the experimental samples were recorded in the region 400–4000 cm^−1^ at a spectral resolution of 4 cm^−1^ [23].

### 2.6. Polarizing Microscope Analysis

Fractured samples were recovered from the transverse strength test and analyzed using a polarizing microscope (LEICA DM 2500P, Germany) samples were stretched out on a glass slide and investigated using optical mineralogic microscope with cross-polarized light, Laboval 2 with an 8-megapixel Samsung digital camera (Samsung, Seoul, Korea) to determine the mode of failure of the different resin materials using for the repair of the nylon base at the fracture surfaces [30].

### 2.7. Statistical Analysis

Statistical analyses were performed using SPSS (Statistical Package for Social Sciences, version 24) computer software. Descriptive statistics including mean values of transverse strength are presented in Table 2. The homogeneity of variances was confirmed by the Levene test. One-way analysis of variance (ANOVA) was used to compare means among all groups (resin material, curing technique, and chemical surfactants), and Sidak's multiple comparisons test was utilized to explore the significance among different groups.

## 3. Results

### 3.1. Fourier Transform Infrared Spectroscopy

**Figure 1:**
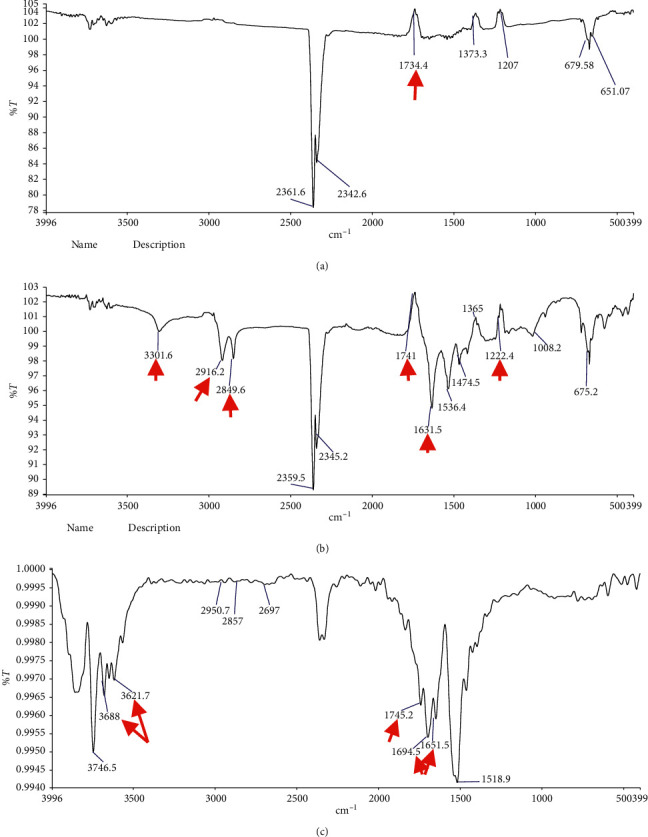
Fourier transform infrared spectroscopy of (a) light-polymerized, (b) Ivomate autopolymerized, and (c) microwave heat-polymerized samples.

In Figures 1(a)–1(c), the spectrum A (light + polyamide nylon base) shows peaks of PMMA compound at 1734 cm^−1^ which signifies C=O stretches of the ester group. However, no peaks were detected for the polyamide nylon. On the contrary, the spectrum B (Ivo + polyamide nylon base) shows two types of C=O stretches at 1741 and 1631 cm^−1^ which correspond to the presence of ester and amide functional groups, respectively. Moreover, the N-H stretching peak appeared at 3301 cm^−1^ that confirms the presence of an amine. This was associated with the C-H aliphatic stretches at 2916 and 2849 cm^−1^, and a C-O stretching peak at 1222 cm^−1^. These results indicated the successful incorporation of both light and Ivo PMMA with polyamide nylon base. The microwave-curing technique was also applied to investigate the two components (PMMA and poly nylon) and to study the effect of microwave on the chemical functional group, as shown in spectrum C. C=O stretching peaks of ester and amide appeared at 1745, 1694, and 1651 cm^−1^. Also, new N-H amide and O-H hydrogen bond peaks were seen at 3688 and 3621 cm^−1^. These peaks demonstrated the increase in the bond strength of microwave repair in comparison to the other repair techniques. In conclusion, the presence of hydrogen bonding which comes from the ester and amide groups in the microwave-curing technique demonstrated significant enhancement of the bond strength.

### 3.2. Transverse Strength Test

**Figure 2:**
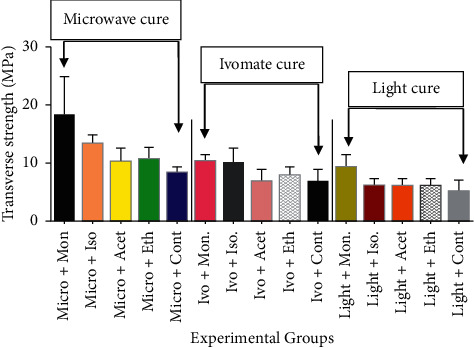
Mean and standard deviations of transverse strength values of experimental and control groups.

The mean values and standard deviation of the transverse strength of the nylon base repaired with different materials, techniques, and surface treatments assessed in this study are presented in Table 2 and Figure 2. The findings of the current study indicated that the transverse strength values of the tested samples increased after chemical surface treatment in comparison to the control group. These results are consistent with previous studies presented by Suad and Intisar [13], Firas et al. [31], and Kümbüloğlu et al. [32]. The transverse strength of the adhesive bond pretreated using monomer was 115.93%, 46.33%, and 69.17% which were fabricated using micro, Ivo, and light PMMA, respectively. The transverse strength of the adhesive bond pretreated using isopropyl was 59.03%, 30.04%, and 17.74% which were fabricated using micro, Ivo, and light PMMA, respectively. The transverse strength of the adhesive bond pretreated using acetone was 22.55%, 3.94%, and 12.18% which were fabricated using micro, Ivo, and light PMMA, respectively. The transverse strength of the adhesive bond pretreated using ethyl acetate was 28.80%, 13.82%, and 9.85% which were fabricated using micro, Ivo, and light PMMA, respectively. For surface treatments using different chemicals, the highest transverse strength values were obtained by monomer treated samples (18.29 N/mm^2^) in comparison to the other groups. While for the curing techniques, the highest transverse strength values of the repaired samples were obtained by micro, followed by Ivo, and then the light-curing technique. The results in Table 3 revealed that the transverse strength of the control (non-surface treated) light-curing groups was lower than those of other surface-treated groups, but the difference was not statistically significant, while a significant difference between the transverse strength of the control group fabricated using microwave curing with the ethyl acetate-treated group was noted. Also, a significant difference between the transverse strength of the control group fabricated using Ivomate curing with the acetone-treated group was noted. The means were found to be significantly different with a *p* value < 0.0001 as listed in Table 4. The results of this study agree with the hypothesis that the physical and chemical properties of denture base materials, also surface treatments have a statistically significant effect on the bond strength of the resin used for the repair.

### 3.3. Polarizing Microscope Analysis

**Figure 3:**
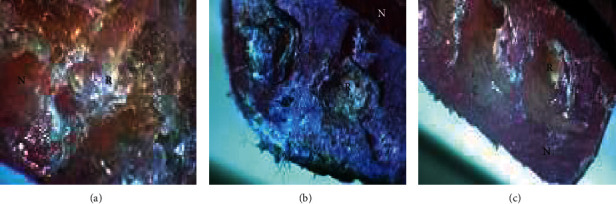
Polarizing microscope images of (a) microwave heat-polymerized, (b) Ivomate autopolymerized, and (c) light-polymerized repaired samples showing the nylon base (N) and the repair materials (R).

**Figure 4:**
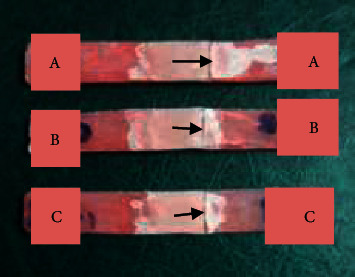
Nylon denture base was repaired by (a) microwave heat-polymerized, (b) Ivomate autopolymerized, and (c) light-polymerized samples. The black arrows indicate the fracture lines.

The polarizing microscope images revealed a cohesive fracture within repair materials rather than adhesive in the interface as seen in Figure 3. This finding is consistent with the results obtained by Rached et al. [29] which revealed a high incidence of mixed fractures (72.2% in an interface and repair material) when repairing specimens with an autopolymerizing, microwave-polymerized, and conventional heat-polymerized acrylic resin. Moreover, Figure 4 exhibits fracture at the junction of the nylon base with microwave-, Ivomate-, and light-polymerized PMMA repair materials rather than through the center of the repaired part.

## 4. Discussion

The fabrication of a new denture is an expensive and time-consuming procedure. For this reason, the decision to repair a denture, whether as an interim or definitive treatment, is a common management plan [33]. The target is to restore the actual strength of the denture and to avoid any further fracture propagation. Nevertheless, the fracture of the repaired samples often occurs at the interface junction of the original base and repair materials rather than through the center of the repair where the load is directed. This finding reveals that the interface of the old and new materials is the site of stress concentration during the transverse strength testing, regardless of the technique and the material used to perform the repair [14]. The validity of the three-point bending test has been closely correlated with the mode of failure of the tested samples [10, 18, 34]. Therefore, the three-point bending test was utilized for this study. Bond strength is the force in demand to break the bond together with failure occurring in or close the adhesive-adherence interface [35].

Having clarified the inherent characteristics of the nylon denture base, the next logical step is to investigate if nylon dentures can be repaired using heat-, auto-, and light-polymerized repair materials. Therefore, the objective of this study was to investigate the bonding strength of the nylon base to various repair materials employing the transverse strength test.

In the present study, the tested samples exhibited cohesive fracture within repair materials at the junction of the nylon base and repair materials rather than through the center of the repaired zone. This fracture could be explained by the differences related to the strength and numbers of a primary (covalent) bond between the atoms and secondary (hydrogen) bonds between adjacent chains, along with the absence of the crosslinking agent in the nylon structure [36]. Furthermore, the low bond strength values compared to the conventional denture base may be attributed to the differences in the polymerization reaction during processing, which is condensation polymerization in the nylon base in comparison with the addition polymerization in the repair materials [37]. On the other hand, nylon particles are more packed (less intermolecular spaces) with less water diffusion where the polymer chains do not contain side groups. This explanation is consistent with Yota [25] who stated that nylon is a highly chemical resistant material (less solubility in solvents) and high heat resistance due to a high degree of crystallinity compared to amorphous acrylic resin.

The findings of the current study indicated that chemical surface treatments and repairing techniques could improve the bonding strength of the nylon denture base polymer to heat-, auto-, and light-polymerized resins that can be utilized for the repair and adjustment of nylon dentures.

Higher transverse strength values were obtained for the microwave-polymerized repair samples (for control and chemical surface treatments) compared to the other sample bases. This finding was consistent with the results of the FTIR test that revealed the presence of hydrogen bonding from the ester and amide groups in the microwave repair samples which are known to significantly enhance the bond strength. As well, it may be attributed to the lower residual monomer levels in microwave-polymerized acrylic compared to conventionally polymerized resins. Also, it has been suggested that the residual monomer levels have a negative effect on the strength of repaired samples as explained in the previous study by Yunus et al. [38]. It seems that the microwave curing lies in the way the monomer molecules are positively moved by a high-frequency electromagnetic field into the network of the polymer molecules; these movements mostly due to internal heat produced during polymerization as explained by De Clerck [39]. Further support for the relatively lower monomer content in the microwave-polymerized PMMA-based polymer comes from the higher strength [40]. Another explanation of the lower monomer content was due to the effect of heat and high-frequency electromagnetic field that might improve the diffusion of the residual monomer to the active sites of the polymer chain, thereby promoting further polymerization that increases the degree of conversion and, subsequently, increased the bond strength between both materials [41]. Other studies revealed that the microwave-cured resin exhibits less porosity as compared to the autopolymerized resin because the heat needed to break the benzoyl peroxide particles into free radicals is generated inside the resin; thus, the penetration capability will be improved [33, 42]. On the other hand, the higher degree of temperature reached during polymerization may lead to more softening of the surface layer of the flexible samples and more penetration of repaired material into the surface layer. Our findings are in agreement with the results obtained by Firas et al. [31] which showed the highest transverse strength values for heat-cured repair materials as compared to cold and light-cured acrylic repair materials.

The transverse strength values obtained for Ivomate repair samples were lower than microwave repair samples for control and different chemical surface treatments. This study hypothesized that water existing during polymerization could reduce the strength of the repair material by decreasing the heat generation associated with the polymerization and it may retard the rate of the polymerization process. Consequently, the monomer may retain the capacity for further diffusion into the denture base resin as explained by Minami et al. [16]. Furthermore, Anusavice [43] reported that the water molecules interfere with the PMMA polymer chains and act as a plasticizer which can adversely affect the strength of the repair material.

The transverse strength values of nylon samples repaired by light curing were lower than that of samples repaired by the micro- and Ivo-curing techniques (for control and different chemical surface treatments), and this result may be attributed to the higher viscosity of light-cured resin which makes the diffusion of the repair material into the nylon base lesser than that shown by other groups which in turn leads to a poor adhesive bond. Also, the light-cured acrylic consists of inorganic fillers with less homogeneity which makes the structural appearance of the material similar to composite and also renders it mechanically brittle. The current study is in agreement with Al-Taie and Khamas, which reported the brittleness of light-cured resin, and it was noted that the light-cured polymerization cannot be performed under pressure as it often led to defects and internal voids [27].

To increase the bond strength between the nylon base and the repair materials, various organic solvents such as monomer, acetone, ethyl acetate, and isopropyl were applied as surface treatment chemicals. Generally, these organic solvents make the repaired surface free from any contamination produced by the cutting process, and also, it may change the morphology and chemical properties of the fractured surface.

In this *in vitro* study, the higher transverse strength values obtained for monomer (MMA) surface-treated samples as compared to other organic solvents may be attributed to the fact that the surface of the dissolved nylon resin depends on monomer interdiffusion, surface swelling, and production of micropores which act as mechanically retentive tags which contribute to the formation of interpenetrating polymer networks during polymerization, hence improving bonding of the repair material to the chemically treated nylon resin surface. MMA liquid acts as a reactive solvent with which makes an interlocking bond with repaired material (chemical retention), especially for heat cure acrylic leading to an increase in the functional sites which in turn produces a stronger transverse bond strength. This finding is in agreement with the previous study by Firas et al. [31].

Isopropyl alcohol is a relatively nontoxic compound which can dissolve polyamide and evaporate quickly as compared to other solvents; hence, it is widely used [13]. This study revealed that there was an increase in transverse strength values after treatment with isopropanol as compared to control groups, and also, it shows higher values when compared with the acetone and ethyl acetate, and this improvement in the strength was probably attributed to the effect of isopropanol to form strong bonds (hydrogen bonds) with a suitable organic compound such as methyl methacrylate, methyl groups in isopropanol which act as electron-releasing agent leading to increase in the electron density on C_2_ in isopropanol, so the electron- negativity on the oxygen atom in C-OH will increase via induction, causing a very strong bond with acrylic [44].

Ethyl acetate is primarily utilized as an organic nonpolymerizable solvent and diluent, being preferred because of its low cost, low toxicity, and agreeable odor. As well, ethyl acetate was an effective cleaning agent; therefore, it is commonly utilized to clean circuit boards and in some nail varnish removers [45]. The present study shows an increase in transverse strength values after treatment with ethyl acetate when compared to the control groups, and this finding may be attributed to the surface treatment with ethyl acetate which causes superficial crack propagation and formation of numerous pits due to the dissolution of nylon base. This increases the mechanical interlocking leading to improved adhesion between repaired surfaces [46]. Another explanation was provided by Shimizu et al. [47] which stated that the dissolution value of ethyl acetate is similar to that of PMMA which allows it to swell the treated surface and permit diffusion of repair resin to the nylon base material.

Acetone is used as a solvent in pharmaceutical industries and also to synthesize methyl methacrylate [45]. Wetting the cutting surface of nylon base with acetone could wash away most of the microdebris and create a sponge-like structure, and these surface modifications will improve the bond strength of the repaired material to the nylon base [11]. The present study also shows an increase in transverse strength values after treatment with acetone as compared to control groups, while these values are lower when compared with the isopropanol and monomer. The lower transverse strength values might be attributed to the trapping of residual acetone between the PMMA which has been documented and explained in a previous study by Memarian and Shayestehmajd [48].

The *in vitro* debonding of repair resin materials from a nylon base can be evaluated by measuring the transverse bond strength, as documented in this study. However, this method did not imitate and simulate the ideal clinical performance, as repaired dentures are exposed to recurrent mechanical stresses during mastication. Also, the sample did not physically simulate the structure of the actual denture construction. Therefore, further assessments are necessary to assess the bonding performance under more closely simulated clinical conditions.

## 5. Conclusion

Polyamide nylon surfaces can be treated using different chemicals, which change the morphology and chemical properties of the surface and promote adhesion. Within limitations of this *in vitro* study, the microwave-polymerized resin was considered as the most effective repair technique along with monomer chemical etchant which creates a tight adhesion between PMMA and nylon denture base in comparison to other groups. On the other hand, the light-polymerized repair technique along with ethyl acetate chemical etchant was the less effective repair technique for the nylon denture base.

## Figures and Tables

**Table 1:** Materials used in this study.

Material	Brand name	Manufacturer	Batch number	Application	Processing method
Polyamide resin (Nylon 12)	Valplast™	Valplast International Corp., NY, USA	QTY: 10	Denture base material	Injection moulding technique; heating to 288°C under pressure of 0.1 MPa. The screwed flask was placed in hot oven at 75°C for 12 min
Heat-polymerized PMMA acrylic resin	Vertex™ Rapid simplified	Vertex, Netherlands	XX131P11	Repair material	Heat processed with a 500 W for 3 min
Autopolymerized PMMA acrylic resin	Vertex™ Castapress	Vertex, Netherlands	XY441P01	Repair material	Dough material cure at 45°C for 15 min under pressure of 2 bars
Light-polymerized acrylic resin	Unifast LC	GC Corp., Japan	Powder: 0712033 Liquid: 0712122	Repair material	The curing cycle was 470 N wave length for 10 min
Methyl methacrylate monomer	Vertex™ Rapid simplified	Vertex, Netherlands	XX131P11	Surface treatment material	Butt joint surface swabbed with monomer for 60 s
Acetone (CH_3_)_2_CO	Acetone	MW:58.08, UK	09200	Surface treatment material	Butt joint surface swabbed with acetone for 30 s
Ethyl acetate (CH_3_COOC_2_H_5_)	Ethyl acetate	MW:88.11, UK	09706	Surface treatment material	Butt joint surface swabbed with ethyl acetate for 120 s
Isopropanol (C_3_H_8_O)	Isopropanol	MW:60.10, UK	09200	Surface treatment material	Butt joint surface swabbed with isopropanol for 5 s

PMMA: poly(methyl methacrylate).

**Table 2:** Mean and standard deviation (SD) of transverse strength of nylon base repaired with different materials, curing techniques, and surface treatments.

Repair technique	Surface treatments	*N*	Mean (N/mm^2^)	SD
Microwave heat-polymerized	Monomer	10	18.29	6.62
	Isopropyl	10	13.48	1.43
	Acetone	10	10.39	2.19
	Ethyl acetate	10	10.92	1.83
	Control (without surface treatment)	10	8.47	0.86

Ivomate autopolymerized	Monomer	10	10.39	1.13
	Isopropyl	10	10.18	2.35
	Acetone	10	7.37	1.47
	Ethyl acetate	10	8.07	1.11
	Control (without surface treatment)	10	7.09	1.82

Light curing	Monomer	10	9.44	2.21
	Isopropyl	10	6.37	0.90
	Acetone	10	6.26	1.02
	Ethyl acetate	10	6.13	1.14
	Control (without surface treatment)	10	5.58	1.61

**Table 3:** Multiple comparison test for the control (non-surface treated) samples with other surface-treated samples using ANOVA with Sidak's test.

Group comparison		Paired differences					*P* value
		Mean difference	Std. deviation	Std. error mean	95% confidence interval for difference		
					Upper	Lower	
Control light	Mon + light	−2.57	2.61	1.06	−5.32	0.17	0.06
	Acet + light	0.00	3.41	1.39	−3.58	3.58	1.00
	Eth + light	0.06	1.80	0.73	−1.82	1.95	0.93
	Iso + light	0.61	0.99	0.40	−0.42	1.65	0.19

Control microwave (micro)	Mon + micro	−1.63	10.94	4.46	−13.11	9.85	0.73
	Acet + micro	3.83	4.57	1.86	−0.95	8.63	0.09
	Eth + micro	2.62	2.53	1.03	−0.02	5.28	0.05*∗*
	Iso + micro	1.91	4.56	1.86	−2.87	6.69	0.35

Control Ivomate (Ivo)	Mon + Ivo	−1.77	3.44	1.40	−5.39	1.84	0.26
	Acet + Ivo	1.91	1.70	0.69	0.11	3.70	0.04*∗*
	Eth + Ivo	1.27	2.60	1.06	−1.46	4.01	0.28
	Iso + Ivo	0.63	5.53	2.25	−5.17	6.44	0.78

Significant difference between groups at *P* < 0.05.

**Table 4:** Results of ANOVA.

Source of variation	Sum of squares	d*f*	Mean square	*F* ratio	Sig. (*P* value)
Between groups	1563	14	111.60	22	*P* < 0.0001
Within group	90.51	9	10.06	2.03	*P* = 0.0405
Residual (random)	622.70	126	4.94		
Total	2276	149			

## Data Availability

The data file of this study is available from the corresponding author upon reasonable request.
